# A road to optimising maternal and newborn quality care measurement for all

**DOI:** 10.1016/S2214-109X(20)30519-2

**Published:** 2021-01-06

**Authors:** Vanessa Brizuela, Özge Tunçalp

**Affiliations:** a The Partnership for Maternal, Newborn and Child Health, World Health Organization, Geneva 1211, Switzerland; b UNDP/UNFPA/UNICEF/WHO/World Bank Special Programme of Research, Development and Research Training in Human Reproduction, Department of Sexual and Reproductive Health and Research, World Health Organization, Geneva 1211, Switzerland

Improvements in maternal and newborn care will continue to fall short if we do not address quality of care. Global frameworks, standards, and measures for quality of maternal and newborn care provide a blueprint for countries to adapt and tailor to their settings.^1^, ^2^ Measuring quality of care is essential to this process. Knowing how to measure and what to measure is equally important.

In *The Lancet Global Health*, Louise Day, Qazi Rahman, and colleagues,^3^ using a large sample of mother–baby pairs receiving care at facilities providing comprehensive emergency obstetric and neonatal care in Bangladesh, Nepal, and Tanzania, assessed the validity of measurement of common facility-based indicators of maternal and newborn care coverage, including some for small and ill newborn babies. The authors highlighted the strengths and weaknesses of two different data collection methods—exit surveys and facility registers— against the gold standard of direct observation, offering a roadmap to improvement that ensures that we are accurately measuring and acting on what matters. This extensive exercise analysed coverage of uterotonics for the prevention of post-partum haemorrhage, early initiation of breastfeeding, neonatal resuscitation, kangaroo mother care, and treatment of neonatal infection.

In general, survey data were more consistent yet were overall less accurate than register data. These findings have important implications, especially considering the reliance on exit surveys in large, facility-based assessments. Previous studies have shown that widely used facility-based surveys are limited with regard to capturing the breadth of quality maternal and newborn care.^4^, ^5^ Given that exit surveys will always be necessary, this study supports the notion that not only do data collection surveys need improvement but also that we need to ensure that women, their companions, and families are empowered, receiving effective communication regarding their care during labour and childbirth, including caesarean sections.

In comparison, Day, Rahman, and colleagues found that the quality and performance of facility registers were highly heterogeneous. In some high-performing hospitals, data were highly accurate and sensitive, whereas in lower-performing hospitals data were less so. While this finding is not novel, it is critical given that the measurements were for common interventions that are central to maternal and newborn health and survival. Because facility registers are commonly used worldwide and have been shown to be capable of capturing most essential indicators,^6^ this finding provides further support for the need for strong health information systems able to capture the right measurements in ways that can alleviate the burden of excess data collection from health-care workers. Registers carry the potential for sustainable, good quality data collection through sound design, thoughtful inclusion of indicators, continuous training, and quality assurance mechanisms—especially as most countries transition to digital platforms. The efforts being made by the Health Data Collaborative might also provide guidance into unified, standardised, and easily reported management systems for health information.

Importantly, the authors found that surveys and facility registers underestimated common maternal and newborn care interventions. How do we, then, use them to assess facility readiness or to inform research, policy, and programming decisions? Relying on women to accurately report interventions that either she or her newborn baby received is questionable. This recall is particularly difficult if it requires an acuity for timing and specificity (was the injection their newborn received an antibiotic or something else?). Trustworthiness of facility registers to capture data systematically is also unequal. For example, the authors were unable to use facility register data to assess administration of antibiotics for the treatment of newborn infections or sepsis—one of the main contributors to newborn death.^7^ And even the gold standard, direct observation, proved hard when the indicator was open to interpretation (eg, does early initiation of breastfeeding mean that the newborn baby was put to the mother's breast soon after birth or that the baby started sucking within 1 h of birth?). We should take heed of the implications of these findings to support and improve fit-for-purpose data collection mechanisms.

One important gap identified in Day, Rahman, and colleagues' study is the measurement of experience of care, where, despite progress in the past 5 years, major gaps exist.^8^ Although essential interventions are important, we know that more is needed to ensure that women and newborn babies survive and thrive. And we also know that many of the measures relating to experience of care might not be reflected in facility registries or might not be properly captured through observation, particularly when related to stigmatising and discriminatory practices. Reports have highlighted the sobering fact that many women are mistreated during childbirth and that newborn babies do not receive all recommended practices.^9^, ^10^ If we continue to set these measures aside when assessing maternal and newborn care, we will keep falling short on our commitment to improve their health and wellbeing.

As we move towards ensuring women and newborn babies receive the optimal care they need and deserve, this study renews our call for better data and better use of the data that are being collected. Health-care workers, as well as mothers and their families, need to see that the collection of time-sensitive data is valuable for health systems to implement new policies, improve services, ensure availability of resources, and provide the training and information necessary to do so accurately.
